# Challenging the gold standard: methods of sampling for microbial culture in patients with chronic rhinosinusitis

**DOI:** 10.1007/s00405-021-06747-z

**Published:** 2021-03-27

**Authors:** Joanna Szaleniec, Agnieszka Gibała, Patryk Hartwich, Karolina Hydzik-Sobocińska, Marcin Konior, Tomasz Gosiewski, Maciej Szaleniec

**Affiliations:** 1 Department of Otolaryngology, Faculty of Medicine, Jagiellonian University Medical College, Jakubowskiego 2, 30-688 Kraków, Poland; 2 Jerzy Haber Institute of Catalysis and Surface Chemistry Polish Academy of Sciences, Niezapominajek 8, 30-239 Krakow, Poland; 3 Department of Molecular Medical Microbiology, Faculty of Medicine, Jagiellonian University Medical College, Czysta 18, 31-121 Krakow, Poland

**Keywords:** Rhinosinusitis, Microbiome, Microbiota, Sampling, Microbiology, Bacteriology

## Abstract

**Purpose:**

Chronic rhinosinusitis (CRS) is a highly prevalent multifactorial disorder. Culture-directed antibiotics are frequently prescribed to patients with CRS and the middle nasal meatus (MM) is traditionally believed to be a representative sampling site of the sinuses as a whole. The purpose of our study was to reevaluate the reliability of the MM as a sampling site in patients with CRS who suffer from impaired drainage from the sinuses to the MM.

**Methods:**

Swabs and tissue biopsies were collected from the MM, maxillary sinus and frontal sinus from 50 patients with CRS. The results of bacterial culture were compared between sampling methods and sites in relation to the patency of the sinus ostia.

**Results:**

782 bacterial isolates were cultured from the samples. Concordant results between the MM and the sinus cavity were noted in 80% of patients for the maxillary sinus, but only 66% for the frontal sinus and 76% for the sinuses a whole. The differences were similarly prevalent in patients with open and occluded sinus ostia. Notably, swabs from all three sites provided representative information in 92% of patients and tissue biopsies did not provide additional information compared to multiple swabs.

**Conclusion:**

The traditional method of sampling from the middle meatus provides inadequate information in 24% of patients with CRS, which may result in inadequate antibiotic therapy and contribute to increasing antibiotic resistance. Additional sampling from the sinuses should be recommended whenever possible, while invasive sampling is not necessary.

**Supplementary Information:**

The online version contains supplementary material available at 10.1007/s00405-021-06747-z.

## Introduction

The role of bacteria in chronic rhinosinusitis (CRS) is unclear, but growing evidence links the disease to bacterial dysbiosis and biofilm formation [1, 2]. The mainstay of therapy in CRS is anti-inflammatory treatment followed by surgery in refractory cases. Antibiotics are currently recommended mainly during exacerbations and in these cases culture directed therapy is most reasonable. In selected cases, macrolides or doxycycline seem to be beneficial, probably due to their anti-inflammatory properties. Nevertheless, antibiotics are frequently prescribed to patients with CRS disregarding these recommendations. Inadequate use of antibiotics for rhinosinusitis is an important factor contributing to the global increase of antibiotic resistance [3].

Bacterial identification techniques used in patients with CRS include conventional culture and molecular methods [1]. Nucleic acid-based studies provide extensive information for microbiome research, but the culture remains irreplaceable for the majority of practical purposes, including pathogen identification and testing for antibiotic resistance. Similarly, isolation of viable bacteria is a prerequisite for in vitro testing of novel antimicrobial therapies such as bacteriophage typing [4].

The determination of the optimal sampling site for patients with CRS is nontrivial. Traditionally, the middle nasal meatus (MM) was presumed to be optimal for this purpose. After the introduction of endoscopes, it replaced the maxillary sinus puncture as the new “gold standard” [5]. However, in CRS, the drainage from the sinuses to the MM is frequently impaired. In addition, occlusion of the sinuses can cause niche-specific differences in the microbiota [6]. As a result, in patients with CRS, the MM seems to be less likely to harbor all the bacterial species from the sinuses. Endoscopic sinus surgery (ESS) provides wide communication between the sinuses and the MM, but it does not necessarily restore adequate mucociliary transport of the secretions to the MM. Moreover, doubt arises whether the bacteria responsible for the recalcitrance of the disease can be identified in superficial swabs without tissue biopsies, because they frequently form biofilms [2], dwell in intramucosal microcolonies or within mucosal cells [7].

The objective of this study was to compare the diagnostic accuracy of the MM swab with other methods of sampling and recommend an optimal method for practical purposes.

Hypotheses:

1. The MM is not a representative sampling site in patients with CRS.
2. The optimal sampling method should be patient-tailored and depend on the patency of the sinus ostia.
3. Tissue biopsies provide additional information compared to noninvasive swabs.

## Materials and methods

### Ethical considerations

The study protocol was approved by the Jagiellonian University Medical College Bioethics Committee (1072.6120.78.2018).

### Study design and participants

This prospective study of diagnostic accuracy has been reported according to the STARD guidelines [8]. Patients undergoing ESS for medically refractory CRS were recruited from the Otolaryngology Clinical Department of the University Hospital in Krakow between October 2018 and June 2019. The diagnosis of CRS was based on the criteria of EPOS 2012 [9]. According to EPOS 2020, most patients presented with primary diffuse CRS [3]. Patients were excluded if they received antibiotics within one month before surgery or met the EPOS 2012 criteria of exclusion from general studies. Fifty consecutive patients who fulfilled the eligibility criteria and gave consent to participate were included in the study. The patency of the sinus ostia was evaluated in the preoperative computed tomography scan that was performed no earlier than 6 months before surgery and verified endoscopically during surgery.

### Specimen collection and microbial identification

**Fig. 1:**
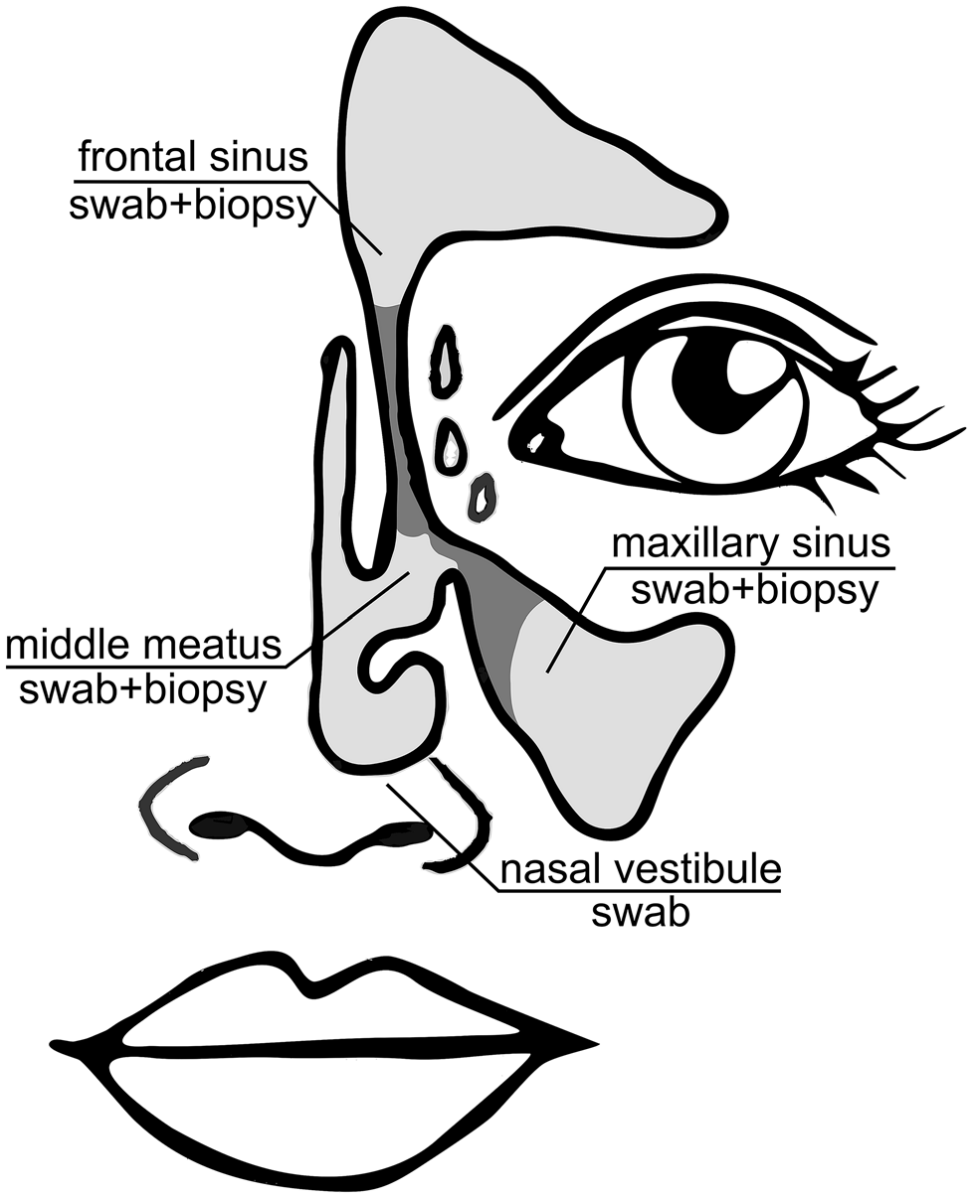
Sampling sites used in the study: pairs of swabs and biopsies were taken from the middle meatus, maxillary sinus and frontal sinus and a reference a swab was taken from the nasal vestibule

Samples were collected during ESS. One swab was taken from the nasal vestibule. Next, three pairs of specimens (a swab and a tissue biopsy) were taken from the MM, the maxillary sinus and the frontal sinus under endoscopic control (Fig. 1). Contact with other sites was avoided and in case it occurred, the contaminated samples were discarded. Samples from sinuses with blocked or narrow ostia were taken immediately after their surgical opening. Bacterial culture, identification and antibiotic susceptibility testing were performed according to The European Committee on Antimicrobial Susceptibility Testing criteria and previously described procedures [10]. The tissue biopsies were rinsed and crushed in a mortar before further processing.

### Data analysis

To describe if sample A provided complete information about the pathogens in sample B, the concordance of results was defined as follows:

- T( +)—true positive—identical pathogens in samples A and B;
- T(−)—true negative—no pathogens in sample A or B;
- F( +)—false positive—at least one additional pathogen in sample A, but absent in sample B;
- F(−)—at least one pathogen missed in sample A, but present in sample B.

Indeterminate results, where one pathogen was present in sample A and another in sample B were included in the F(−) category because they represented a similar type of error (undetected pathogen). The same species but with different antibiotic resistance mechanisms were defined as different pathogens because their identification would result in different therapeutic decisions. The concordance between sampling options and the sensitivity and specificity of each method was calculated to identify the most accurate index test. The MM swab served as the reference “gold standard”.

### Statistical analysis

The Cochran’s *Q* test was used to assess the differences in the occurrence of each bacterial species between the sampling sites. The Chi-square test was used to determine the association between the size of the sinus ostium and the concordance of culture results. Statistical significance was considered at the 0.05 level.

## Results

**Fig. 2:**
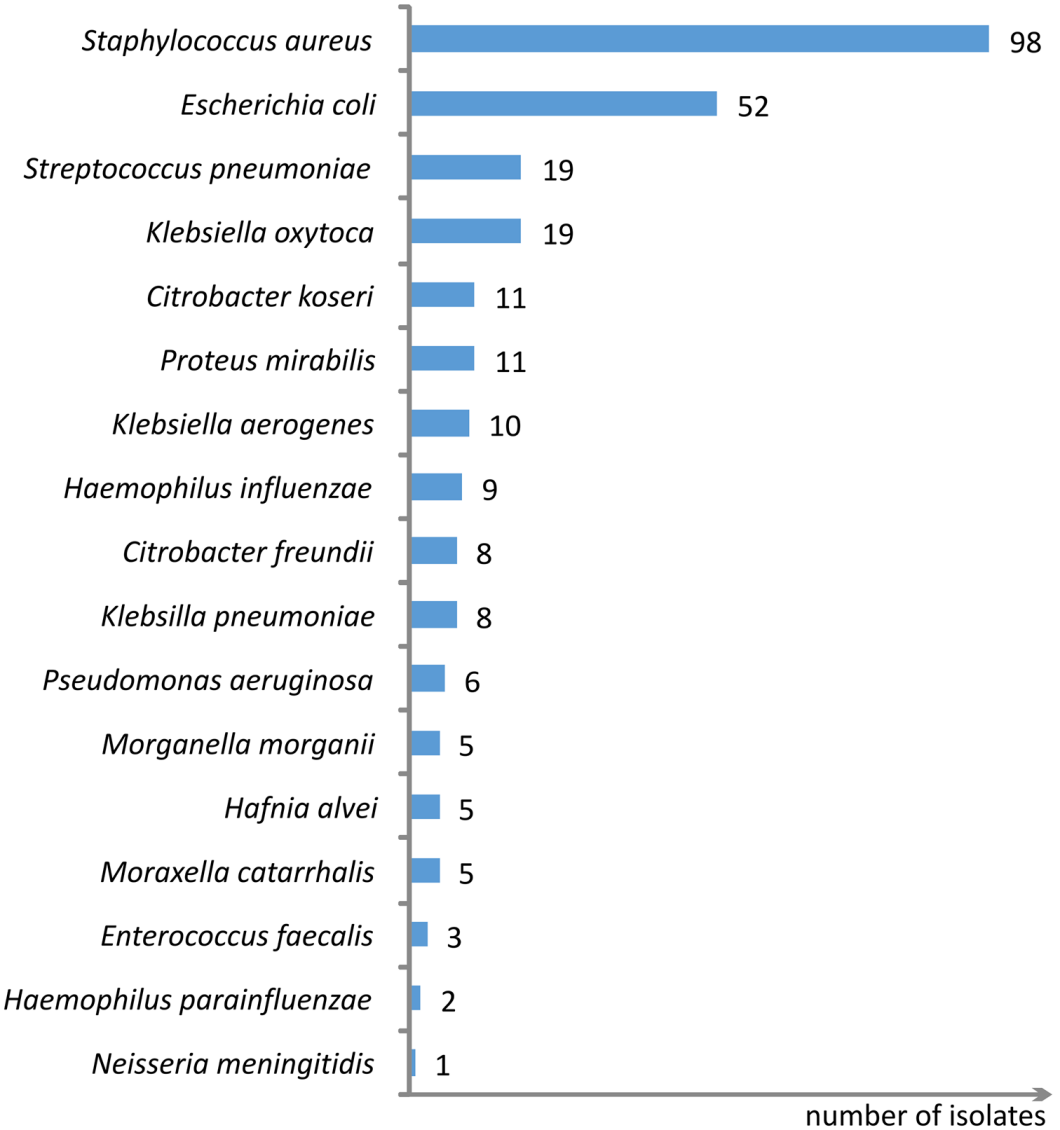
Pathogenic bacterial species identified in the samples (number of isolates), *n* = 272

The patients’ characteristics are shown in Table 1. The flow of participants is illustrated in the supplementary material (Fig. S1). Seven samples from each of the 50 participants generated a collection of 350 samples. A total of 782 bacterial isolates were cultured from the samples (0–5 isolates per sample). Bacteria that are considered non-pathogenic (coagulase-negative staphylococci, most *Corynebacteria*, salivarius group of streptococcus) were identified in 510 isolates. To facilitate comparisons with other studies we analyzed only the species that are classically considered pathogenic (272 isolates—Fig. 2). Detailed information about the bacteria identified in the samples and their antibiotic resistance is openly available in the Mendeley Data [11].

**Table 1:** Patient characteristics (n = 50)

Gender	Female 25 (50%)
Age	19–83 (mean 49)
Nasal polyps	21 (42%)
ESS in the past	24 (48%)
Asthma	20 (40%)
Aspirin-exacerbated respiratory disease	9 (18%)
Allergy	24 (48%)
Gastroesophageal reflux	15 (30%)
Lund–Mackay computed tomography staging score^a^ (total 0–24)	2–24 (mean 13)

^a^Lund VJ, Mackay IS. Staging in rhinosinusitis. Rhinology. 1993;31(4):183–4

### MM swab vs. maxillary sinus swab

As shown in Table 2, concordant results were noted in 80% of patients (sensitivity 93%, specificity 65%). The differences in occurrence between the MM and the maxillary sinus were not statistically significant for any single pathogen. When non-pathogenic bacteria were included in the analysis, the results were identical only in 26% of patients. In 38% of patients, the same non-pathogenic species found in the MM presented different antibiotic resistance mechanisms than the one isolated from the maxillary sinus.

**Table 2:** Ability of the MM swab to detect all the pathogens identified in the maxillary sinus and the frontal sinus (*n* = 50)

		Maxillary sinus (MS) swab	
		POSITIVE (pathogens present)	NEGATIVE (pathogens absent)
The ability of the MM swab to detect all the pathogens identified in the maxillary sinus swab			
Middle meatus (MM) swab	POSITIVE (pathogens detected)	TRUE POSITIVE 25 (50%)	FALSE POSITIVE 8 (16%)
	NEGATIVE (no/not all pathogens detected)	FALSE NEGATIVE 2 (4%)	TRUE NEGATIVE 15 (30%)
		Sensitivity = 93%	Specificity = 65%
		Concordant 40 (80%)	Not concordant 20 (20%)

		Frontal sinus (MS) swab	
		POSITIVE (pathogens present)	NEGATIVE (pathogens absent)
The ability of the MM swab to detect all the pathogens identified in the frontal sinus swab			
Middle meatus (MM) swab	POSITIVE (pathogens detected)	TRUE POSITIVE 21 (42%)	FALSE POSITIVE 9 (18%)
	NEGATIVE (no/not all pathogens detected)	FALSE NEGATIVE 8 (16%)	TRUE NEGATIVE 12 (24%)
		Sensitivity = 72%	Specificity = 57%
		Concordant 33 (66%)	Not concordant 17 (34%)

TRUE POSITIVE—identical pathogens detected in MM and the sinus swab; FALSE POSITIVE—a pathogen detected in MM swab but absent in the sinus swab; FALSE NEGATIVE—a sinus pathogen missed in the MM swab, TRUE NEGATIVE—no pathogens in the MM or the sinus swab. True-positive and true-negative results were reported as concordant

### MM swab vs. frontal sinus swab

The ability of the MM swab to detect frontal sinus pathogens was lower than for the maxillary sinus (Table 2). Concordant results were noted in 66% of patients (sensitivity 72%, specificity 57%). Again, there were no statistically significant differences in the occurrence of any of the pathogenic species. When non-pathogenic species were included, identical results were noted only for 22% of patients. 40% of study participants carried the same non-pathogenic species but with different antibiotic resistance in the MM and the frontal sinus.

### MM swab vs. maxillary sinus swab—the influence of the maxillary ostium size

To study the influence of the maxillary ostium size on the sampling results, we divided the patients into three subgroups: the maxillary ostium was blocked in 46%, narrow in 30% and wide (previously surgically enlarged) in 24% of patients. Statistical analysis proved that the ostium patency or size did not influence the concordance of samples taken from both sides of the ostium.

### MM swab vs. frontal sinus swab—the influence of the frontal ostium size

Almost all of the patients (92%) presented with blocked frontal sinus ostia. Again, there were no statistically significant differences in the concordance of culture results between patients with patent and blocked ostia.

### Swab vs tissue biopsy from the same site

Results of the paired analysis of 150 swabs versus 150 biopsies from the same locations were shown in the supplementary material (Table S1). Identical results were noted in 72% of sample pairs. In 18% of pairs, more pathogens were cultured from the swab, while in 10% a biopsy provided more information. Swabs detected biopsy pathogens with 72% sensitivity and 61% specificity.

### MM swab vs. all samples from the patient

In this analysis, the results were counted as concordant if all of the pathogens identified in the 3 swabs and 3 biopsies from the patient (MM, maxillary sinus and frontal sinus samples) were present in the MM swab. The ability of a MM swab to detect all the pathogens cultured from the patient was noted in 76% of cases (sensitivity 68%), while in the remaining 24% one or more pathogens were missed by a MM swab.

### Nasal vestibule swab vs. all samples from the patient

A similar analysis showed that the nasal vestibule swab detected all the pathogens from the MM and sinus samples only in 52% of patients.

### 3 swabs (MM, maxillary and frontal) vs. all samples from the patient

As mentioned above, some pathogens present in biopsies were absent in swabs from the same site. Unexpectedly, these pathogens were very likely to be found in swabs from other locations. Therefore, we conducted an additional analysis to find out if a series of 3 swabs was able to detect all the pathogens cultured from the patient. We found that this assumption was true for 92% of our study group (sensitivity 89%). Therefore, we propose multiple swabs as a reliable index test to detect sinonasal pathogens. A detailed comparison of multiple swabs versus MM swab is shown in Table 3.

**Table 3:** Comparison of the MM swab (reference “gold” standard) and multiple swabs (index test) for the detection of all the pathogens cultured from the patient’s swabs and biopsies (*n* = 50)

		All samples from the patient (3 swabs + 3 biopsies)	
		POSITIVE (pathogens present)	NEGATIVE (pathogens absent)
The ability of the MM swab to detect all the pathogens cultured from the patient			
MM swab	POSITIVE (pathogens detected)	TRUE POSITIVE 26 (52%)	FALSE POSITIVE N/A
	NEGATIVE (no/not all pathogens detected)	FALSE NEGATIVE 12 (24%)	TRUE NEGATIVE 12 (24%)
		Sensitivity = 68%	Specificity = 100%
		Concordant 38 (76%)	Not concordant 12 (24%)

		All samples from the patient (3 swabs + 3 biopsies)	
		POSITIVE (pathogens present)	NEGATIVE (pathogens absent)
The ability of the 3 swabs (MM, maxillary and frontal) to detect all the pathogens cultured from the patient			
3 swabs (MM, maxillary, frontal)	POSITIVE (pathogens detected)	TRUE POSITIVE 34 (68%)	FALSE POSITIVE N/A
	NEGATIVE (no/not all pathogens detected)	FALSE NEGATIVE 4 (8%)	TRUE NEGATIVE 12 (24%)
		Sensitivity = 89%	Specificity = 100%
		Concordant 46 (92%)	Not concordant 4 (8%)

Reference standard: TRUE POSITIVE—the MM swab detected all the pathogens identified in the patient’s samples, FALSE POSITIVE—not applicable (the MM swab was included in the set of all samples, so it was not possible to detect a pathogen in the MM that was absent in the whole set; for the same reason the test has 100% specificity), FALSE NEGATIVE—a pathogen from any other sample missed in the MM swab, TRUE NEGATIVE—no pathogens in the MM swab or any other sample

Index test: TRUE POSITIVE—multiple swabs detected all the pathogens identified in the patient’s samples, FALSE POSITIVE—not applicable (the MM, maxillary and frontal swabs are included in the set of all samples, so it is not possible to detect a pathogen in these swabs that is absent in the whole set; for the same reason the test has 100% specificity), FALSE NEGATIVE—a pathogen from any other sample missed in multiple swabs, TRUE NEGATIVE—no pathogens in the MM, maxillary and frontal swabs or any other sample

True-positive and true-negative results were reported as concordant

## Discussion

### Comparison of sampling sites

The results of culture from the MM and maxillary swabs in our study showed 80% concordance, which is comparable with the results reported by the other authors [5, 6, 12–27]. A detailed summary of 18 studies on the subject is presented in the supplementary material (Table S2). Classically, only the maxillary sinus was taken into account in such analyses. Nevertheless, the presumption that all the sinuses harbor identical microbiota was proven wrong [18]. Moreover, compared to other sinuses, the maxillary sinus is rarely the point of origin of serious complications [3]. We showed that the MM as a sampling site is much less representative of the frontal sinus than of the maxillary sinus. Therefore, even if the results of MM and maxillary cultures are fairly concordant, this finding is not sufficient to claim that the MM is representative of the patient's entire sinonasal microbiota.

The differences between the MM and the sinuses are even greater if the analysis includes the traditionally ignored “commensal microbiota”. Interestingly, the same non-pathogenic species often showed different antibiotic resistance in two adjacent locations. These observations support the assumption that the MM and the sinuses constitute different niches with specific microenvironments.

To compare our findings with those of other authors it is important to note the differences in defining the term “concordance”. In various studies, the results were considered satisfactory if at least one species was identical in both samples [13, 20], the MM swab identified the predominant pathogen [14] or correctly detected only the “acute pathogens” (*H. influenzae*, *M. catarrhalis*, *S. pneumoniae*) regardless of the presence of other species [15]. In our study, we interpreted the results from the perspective of their clinical implications. Only if the pathogens identified in two samples were identical, we described them as concordant. Failure to detect even a single bacterial strain may lead to improper choice of antibiotics and increasing resistance without benefit for the patient. Our more rigorous definition results in a lower percentage of “concordant” results than reported in most publications.

Due to the heterogeneity of bacteria isolated from the patients and high interpatient variability, the differences between sampling sites are notoriously difficult to capture in statistical analyses. Probably much larger and more homogenous study groups would provide more reliable results.

### Relationship between the patency of sinus ostium and reliability of the MM swab

We initially hypothesized that sampling methods should be patient-tailored (additional sampling from the sinus recommended only if the ostium is blocked). These assumptions were supported by the findings of Kim et al*.* that the size of maxillary antrostomy influences the microbiome composition, although, the study did not compare the similarity between the sinus and the MM [28]. Therefore, in our study, we compared unoperated patients with narrow or blocked ostia with previously operated individuals who had wide sinus openings. Surprisingly, our study showed that the evolution of distinct microbial communities was not limited to anatomically separated niches. This result can be explained by impaired mucociliary transport and stasis of secretions that may persist after surgery. Therefore, sampling from multiple sites is reasonable in all patients with CRS and not only those with blocked ostia.

### Comparison of sampling techniques

A paired analysis of swabs and biopsies from the same locations showed that the concordance of results was relatively low and it was not possible to prove the superiority of either sampling method. The other authors who studied the subject in smaller groups of patients reported contradictory results [7, 24, 25, 29, 30]. More details about previous studies can be found in the supplementary material (Table S3).

In our study, it was striking that usually the pathogens detected in tissue samples were also found in the swabs, but not necessarily from the same location. This observation indicated that multiple swabs may provide sufficient information about the sinonasal microbiota.

### Middle meatus and nasal vestibule swabs versus multiple swabs

The swab from the nasal vestibule was taken to verify the possible contamination of deeper samples. It was concordant with sinonasal swabs only in 52% of patients, which supports the universal opinion that it is useless to diagnose the etiology of sinus infections. The MM swab correctly detected all pathogens culturable from the patient in 76% of patients. Taking multiple swabs (from the MM, maxillary sinus and frontal sinus) improved the reliability of testing and allowed for the detection of all culturable pathogens in 92% of patients. It suggests that a swab from one site may capture pathogens that in other sites are too firmly attached to the mucosa to be sampled by swabbing. Besides, multiple sampling compensates for the inaccuracy of culture as a bacterial identification method.

### Suggested recommendations

Clinicians and researchers should be aware of the failure rates of single-site versus multiple-site sampling and choose a method that is adequate for their particular purposes. For certain areas of research, a MM sample can be sufficient, because it is roughly representative of the patient’s unique microbiota in comparison to other individuals [6]. Nevertheless, if the goal is to capture all sinonasal pathogens that may play a role in the patient’s disease, the MM swab may be seriously misleading. In our opinion, multiple-site sampling should be recommended in the office whenever the patient’s anatomy allows for it and during surgery. It is particularly important if previous antibiotic therapy failed, in immunocompromised patients or in case of complications.

### Limitations

1. The study served a practical purpose (to determine an optimal method of sampling for culture) and the recommendations cannot be extrapolated to molecular studies.
2. The samples in our study were obtained from patients with CRS. In patients with acute rhinosinusitis or exacerbations of CRS more secretions drain from the sinuses and the culture might be more accurate if the swab is taken from the mucopus emanating from the sinus to the middle meatus [14].
3. The study protocol did not include anaerobic cultures, so the results apply only to bacteria that can be cultured in aerobic conditions.
4. Sinus sampling was limited to the maxillary and frontal sinus.

## Conclusion

1. The MM swab is not as representative of sinonasal microbiota as it is generally assumed. Sampling only from the MM may result in missing important pathogens in one out of four patients with CRS.
2. Wide sinus ostium does not imply greater similarity of the microbiota between the sinus and the MM.
3. Invasive sampling (tissue biopsy) usually does not provide additional information compared to multiple swabs.
4. Swabs from multiple locations (middle meatus, maxillary sinus and frontal sinus) provide comprehensive information about the patient’s culturable pathogens in 92% of cases.

## Supplementary Information

Below is the link to the electronic supplementary material.

Supplementary file1 Fig S1 The flow of participants (JPG 382 KB)

Supplementary file2 (DOCX 12 KB)

Supplementary file3 (DOCX 15 KB)

Supplementary file4 (DOCX 12 KB)

## Abbreviations

CRS: Chronic rhinosinusitis
MM: Middle nasal meatus
ESS: Endoscopic sinus surgery

## Declarations

### Availability of data and material

The data that support the findings of this study are openly available in: Szaleniec, Joanna; Gibała, Agnieszka; Hartwich, Patryk; Hydzik-Sobocińska, Karolina; Konior, Marcin; Stręk, Paweł; Gosiewski, Tomasz; Szaleniec, Maciej (2020), “Bacterial isolates cultured from various sampling sites in patients with chronic rhinosinusitis”, Mendeley Data, V1, https://doi.org/10.17632/ps94fp4ngn.1.
