# Supplementary figures and images for: Challenging the gold standard: methods of sampling for microbial culture in patients with chronic rhinosinusitis
Source: Eur Arch Otorhinolaryngol. 2021 Mar 27;278(12):4795–803. doi: 10.1007/s00405-021-06747-z (PMC8553703; doi:10.1007/s00405-021-06747-z)

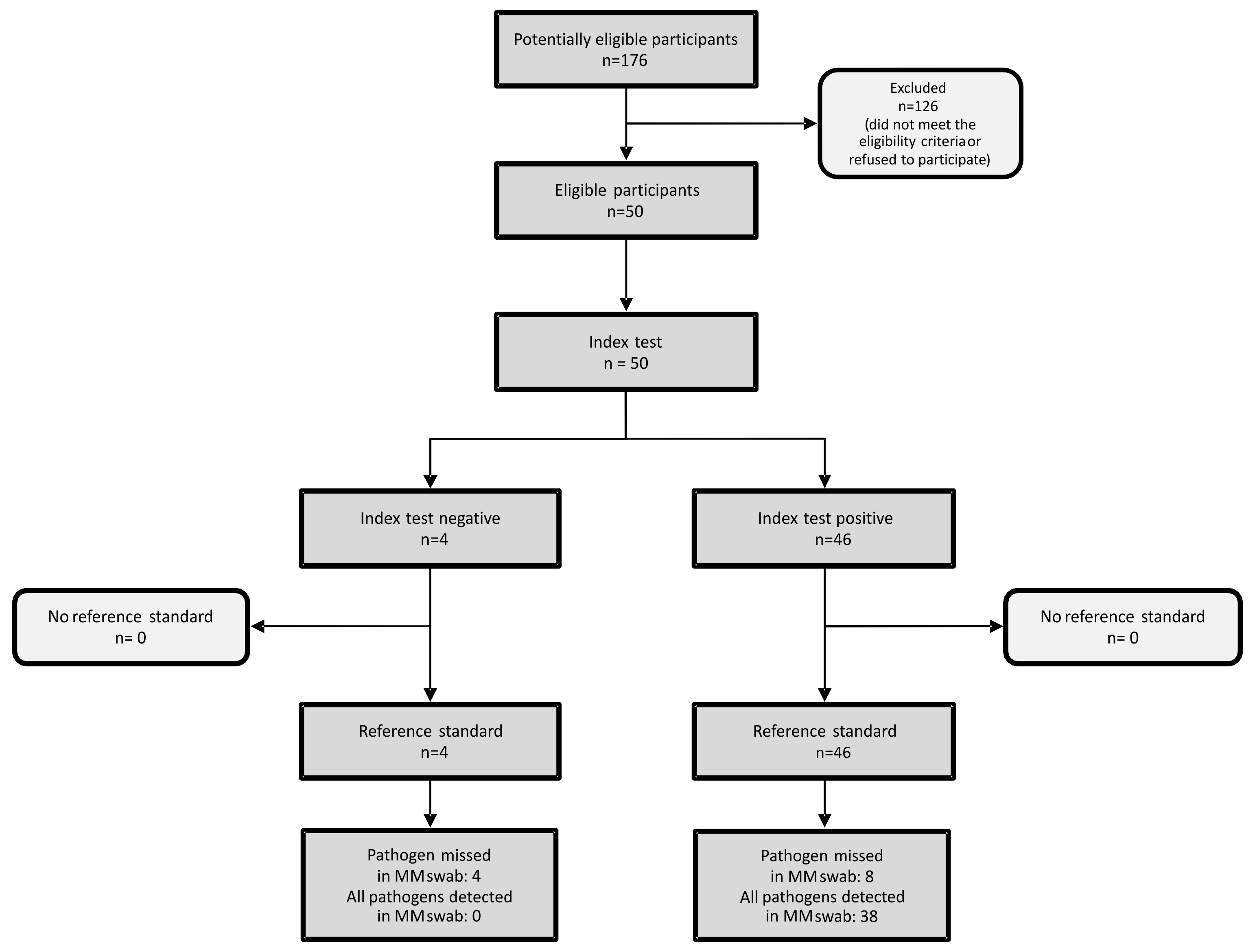

Supplement: Supplementary file 1 — Supplementary file1 Fig S1 The flow of participants (JPG 382 KB) [file 405_2021_6747_MOESM1_ESM.jpg]
