# Supplementary material for: Challenging the gold standard: methods of sampling for microbial culture in patients with chronic rhinosinusitis
Source: Eur Arch Otorhinolaryngol. 2021 Mar 27;278(12):4795–803. doi: 10.1007/s00405-021-06747-z (PMC8553703; doi:10.1007/s00405-021-06747-z)
Supplement: Supplementary file 2 — Supplementary file2 (DOCX 12 KB) [file 405_2021_6747_MOESM2_ESM.docx]

**Table S1. The ability of the swab to detect all pathogens identified in the biopsy from the same site (n = 150).**

TRUE POSITIVE - identical pathogens in swab and biopsy, FALSE POSITIVE - a pathogen in swab but not in a biopsy, FALSE NEGATIVE - a biopsy pathogen missed in the swab, TRUE NEGATIVE - no pathogens in swab or biopsy. True positive and true negative results were reported as concordant.

| **The ability of the swab to detect all pathogens identified in the biopsy from the same site** | | | |
| --- | --- | --- | --- |
|  | | **biopsy from the same site** | |
|  |  | **POSITIVE**  **(pathogens present)** | **NEGATIVE**  **(pathogens absent)** |
| **swab** | **POSITIVE**  **(pathogens detected)** | TRUE POSITIVE  65 (43%) | FALSE POSITIVE  27 (18%) |
|  | **NEGATIVE**  **(no / not all pathogens detected)** | FALSE NEGATIVE  15 (10%) | TRUE NEGATIVE  43 (29%) |
|  | | **SENSITIVITY = 81%** | **SPECIFICITY = 61%** |
|  |  | **CONCORDANT**  **108 (72%)** | **NOT CONCORDANT**  **42 (28%)** |
