# Supplementary material for: Challenging the gold standard: methods of sampling for microbial culture in patients with chronic rhinosinusitis
Source: Eur Arch Otorhinolaryngol. 2021 Mar 27;278(12):4795–803. doi: 10.1007/s00405-021-06747-z (PMC8553703; doi:10.1007/s00405-021-06747-z)
Supplement: Supplementary file 3 — Supplementary file3 (DOCX 15 KB) [file 405_2021_6747_MOESM3_ESM.docx]

**Table S2. Studies comparing sampling from various locations.**

| **Study** | **microbial identification method** | **number of participants** | **health status^a^** | **sampling sites^b^** | **authors' conclusions^c^** | **recalculated % of identical results between the MM and the sinuses (for pathogens)^d^** |
| --- | --- | --- | --- | --- | --- | --- |
| Brook et al. (1997) (5) | aerobic and anaerobic culture | 6 | CRS, ARS | MM, MS | concordance in the type and concentration of organisms in all cases | 67% |
| Gold et al. (1997) (12) | aerobic culture | 18 | CRS, only with purulent secretions in the MM, during antibiotic therapy | MM, MS (mucus trap) | exact correlation: 85,7% | 85,7% |
| Klossek et al. (1998) (13) | aerobic and anaerobic culture | 65 | CRS without exacerbation | nasal cavity (MM, FR, SR), MS | results agreed in 74% of cases | 74% |
| Vogan et al. (2000) (14) | Culture | 13 | ARS | MM, MS | identification of predominant pathogen in MM: 93.8% | 69% |
| Talbot et al. (2001) (15) | aerobic culture | 46 | ABRS (maxillary sinusitis) | MM, MS | accuracy 58,7% (for *H. influenzae*, *M. catarrhalis* or *S. pneumoniae* 89.1%) | 55% (for available data) |
| Kountakis et al. (2002) (16) | Culture | 18 | ARS in intensive care | MM, MS (lavage aspirate) | the same pathogen in 21% of cases | 16% |
| Jiang et al. (2002) (17) | Culture | 186 | CRS | MM, ES | results differed in 50,5% of patients | 49,5% |
| Brook (2004) (18) | aerobic and anaerobic culture | 80 | ARS, CRS | 2-4 sinuses (not the MM) | 19-56% of the isolates present only in a single sinus | n/a |
| Dubin et al. (2005) (19) | Culture | 187 (meta-analysis) | ARS, CRS | MM, MS | accuracy per isolate 82%, per culture 73% | 73% |
| Benninger et al. (2006) (20) | aerobic culture | 7 (+ meta-analysis) | ABRS | MM, MS | accuracy (with meta-analysis) 87% | 57% |
| Araujo et al. (2007) (21) | aerobic and anaerobic culture | 16 | CRS, only with secretions in the MM | MM, MS | 75% of the germs the same in both cultures | 75% |
| Yan et al. (2013) (22) | 16S rRNA sequencing | 12 | healthy | AN, MM, SR | MM and SR nearly identical, AN - different | n/a |
| Biswas et al. (2015) (23) | 16S rRNA sequencing | 6 + 9 | healthy + CRS | AN, IT, MM | microbial community structure did not differ significantly between sites | n/a |
| Joss et al. (2016) (24) | 16S rRNA sequencing | 22 | CRS | AN, MM, MS, ES, SS, FS | substantial variation (mainly in abundance) of bacterial communities between sinuses in some patients, the higher distance between sinuses and nostril | n/a |
| Ramakrishnan et al. (2017) (6) | 16S rRNA sequencing | 8 | CRS, without previous surgery | AN, MM, MS, FS, SS, NP | MM is a fair representation of the underlying sinuses (for longitudinal studies) | n/a |
| Koeller et al. (2018) (25) | aerobic and anaerobic culture, 16S rRNA sequencing | 3 + 15 | healthy, CRS | MM, sinuses (various sites) | some species only detected in one or two samples, no systematic difference between 3 sites of sampling | n/a |
| Copeland et al. (2018) (26) | 16S rRNA sequencing | 12 + 21 | healthy, CRS | AN, MM, MS, ES, SS | sinuses more similar to each other than the MM to the sinuses (MM is not a representative sampling site) | n/a |
| De Boeck et al. (2019) (27) | 16S rRNA sequencing | 100 + 190 | healthy, CRS | AN, NP, MS, ES | strong continuity for the microbiome in different niches | n/a |

^a^ Health-status: ARS - acute rhinosinusitis, ABRS - acute bacterial rhinosinusitis, CRS - chronic rhinosinusitis.

^b^ Sampling sites: AN - anterior nares (nasal vestibule), MM - middle meatus, MS - maxillary sinus, ES - ethmoid sinus, FS - frontal sinus, FR - frontal recess, SS - sphenoid sinus, SR -sphenoethmoidal recess, IT - inferior turbinate, MT - middle turbinate, NP - nasopharynx. Unless stated otherwise, the sampling methods were: puncture with aspiration from the MS, swab from other locations.

^c^ Conclusions as reported by the authors.

^d^ The definition of "concordant results" varied between studies, so the results were recalculated according to our interpretation principles (see "Material and methods") to facilitate comparisons with our results. Therefore, the values may differ from those reported by the authors. Where possible, only samples with identical pathogens were counted as "concordant". Following most authors' assumptions, putative nonpathogenic species were excluded from the calculations. Recalculation was not feasible for studies that used molecular methods.
