# Supplementary material for: Challenging the gold standard: methods of sampling for microbial culture in patients with chronic rhinosinusitis
Source: Eur Arch Otorhinolaryngol. 2021 Mar 27;278(12):4795–803. doi: 10.1007/s00405-021-06747-z (PMC8553703; doi:10.1007/s00405-021-06747-z)
Supplement: Supplementary file 4 — Supplementary file4 (DOCX 12 KB) [file 405_2021_6747_MOESM4_ESM.docx]

**Table S3. Studies comparing sampling techniques.**

| Niederfuhr (2009) (29) | nasal lavage, biopsy | aerobic and anaerobic culture | 21 + 44 | healthy, CRS | ES | in 35% of patients, pathogenic bacteria found only in nasal lavage and not in biopsy |
| --- | --- | --- | --- | --- | --- | --- |
| Bassiouni et al. (2015) (30) | swab, biopsy | 16S rRNA sequencing | 6 | CRS | ES | no significant difference between swab and biopsy (strong correlation except for 1 patient) |
| Kim et al. (2015) (7) | swab, biopsy | 16S rRNA sequencing | 9 | CRS | MM | significant differences in bacterial composition between swab and biopsy (more bacteria from tissue samples) |
| Joss et al. (2016) (24) | swab, biopsy | aerobic culture, 16S rRNA sequencing | 22 | CRS | AN, MM, MS, ES, SS, FS | results from swab and biopsy very similar, differences not significantly different than between sinuses |
| Koeller et al. (2018) (25) | swab, biopsy (culture), biopsy (sequencing) | culture, 16S rRNA sequencing | 3 + 15 | healthy, CRS | MM, sinuses (various sites) | more species cultured from swab |

^a^ Sampling sites: AN - anterior nares (nasal vestibule), MM - middle meatus, MS - maxillary sinus, ES - ethmoid sinus, FS - frontal sinus, SS - sphenoid sinus, MT - middle turbinate, NP - nasopharynx.

^b^Most studies used molecular methods, so the recalculation of results provided in Table 1 was not applicable.
